# Patient-specific simulations predict efficacy of ablation of interatrial connections for treatment of persistent atrial fibrillation

**DOI:** 10.1093/europace/euy232

**Published:** 2018-11-23

**Authors:** Caroline H Roney, Steven E Williams, Hubert Cochet, Rahul K Mukherjee, Louisa O’Neill, Iain Sim, John Whitaker, Orod Razeghi, George J Klein, Edward J Vigmond, Mark O’Neill, Steven A Niederer

**Affiliations:** 1 School of Biomedical Engineering & Imaging Sciences, King's College London, St. Thomas' Hospital, Westminster Bridge Road, UK; 2 LIRYC Electrophysiology and Heart Modeling Institute, Bordeaux Fondation, Avenue du Haut-Lévèque, Pessac, France; 3 Western University, London, Ontario, Canada; 4 IMB, Univ. Bordeaux, Talence, France

**Keywords:** Atrial fibrillation, Computer modelling, Phase singularity mapping, Atrial fibrosis, Ablation therapy, Interatrial conduction

## Abstract

**Aims:**

Treatments for persistent atrial fibrillation (AF) offer limited efficacy. One potential strategy aims to return the right atrium (RA) to sinus rhythm (SR) by ablating interatrial connections (IAC) to isolate the atria, but there is limited clinical data to evaluate this ablation approach. We aimed to use simulation to evaluate and predict patient-specific suitability for ablation of IAC to treat AF.

**Methods and results:**

Persistent AF was simulated in 12 patient-specific geometries, incorporating electrophysiological heterogeneity and fibres, with IAC at Bachmann’s bundle, the coronary sinus, and fossa ovalis. Simulations were performed to test the effect of left atrial (LA)-to-RA frequency gradient and fibrotic remodelling on IAC ablation efficacy. During AF, we simulated ablation of one, two, or all three IAC, with or without pulmonary vein isolation and determined if this altered or terminated the arrhythmia. For models without structural remodelling, ablating all IAC terminated RA arrhythmia in 83% of cases. Models with the LA-to-RA frequency gradient removed had an increased success rate (100% success). Ablation of IACs is less effective in cases with fibrotic remodelling (interstitial fibrosis 50% success rate; combination remodelling 67%). Mean number of phase singularities in the RA was higher pre-ablation for IAC failure (success 0.6 ± 0.8 vs. failure 3.2 ± 2.5, *P* < 0.001).

**Conclusion:**

This simulation study predicts that IAC ablation is effective in returning the RA to SR for many cases. Patient-specific modelling approaches have the potential to stratify patients prior to ablation by predicting if drivers are located in the LA or RA. We present a platform for predicting efficacy and informing patient selection for speculative treatments.

**What’s new?**

- We present a platform for generating preliminary evaluation and guidance on patient selection for more speculative treatment approaches, assessed in a pilot virtual clinical study.
- Our study predicts ablation of Interatrial connections (IAC) is effective for many persistent atrial fibrillation (AF) cases in returning the right atrium (RA) to sinus rhythm.
- Simulated IAC ablation is more effective in cases with no LA-to-RA frequency gradient.
- Simulated IAC ablation is less effective in cases with fibrotic remodelling, particularly in the RA.
- Model phase singularity density in the RA is higher pre-ablation in cases for which IAC ablation failed to terminate RA arrhythmia, suggesting this measure may inform whether IAC ablation is likely to be a suitable ablation strategy for a given persistent AF patient.

## Introduction 

Persistent atrial fibrillation (AF) is often treated using catheter ablation therapy, which typically includes electrical isolation of the pulmonary veins (PVs), together with the option of additional ablation lesions. Ablation therapy may also target electrical drivers that sustain the arrhythmia. The recent STAR AF II trial compared different left atrial (LA) ablation approaches for persistent AF patients to find a recurrence rate of >40% at 18 months.^1^ An alternate strategy is to apply catheter ablation to isolate the atria, and prevent electrical connection between the LA and right atrium (RA).^2^ As AF drivers are predominantly located in the LA,^3^ successfully isolating the atria may allow AF to be terminated in the RA, independent of the state of the LA. This would return the ventricles to sinus rhythm (SR), despite the LA remaining in AF. Further, in persistent patients, the LA may be dilated and scarred and as such the LA haemodynamic contribution may not be substantive even in SR, especially in patients who have undergone LA appendage closure. Consequently, LA isolation may functionally be similar to curative LA ablation.^4^

The atrial muscular architecture determines the electrical connections between the RA and LA. Connection sites include: the coronary sinus (CS), the interatrial septum [for example, at the fossa ovalis (FO)], and Bachmann’s bundle (BB). These interatrial connections (IAC) determine the regular SR conduction pattern. Lemery *et al.*^5^ measured atrial activation patterns during SR and demonstrated that conduction occurs over BB in at least 88% of patients, causing the anterior LA to activate significantly earlier than the posterior LA. In addition, CS conduction was measured in 93% of patients.^5^ The length of myocardial tissue in the CS, and the location of LA-CS connection points, is highly variable.^6^ Sites of IAC at the interatrial septum also vary between individuals; Lemery *et al.*^5^ found that there was no electrical conduction through the FO in 69% of patients.

Previous studies have suggested targeting IACs in ablation or surgical treatment for AF. For example, Chauvin *et al.*^7^ suggested a role of the CS in AF and consequently proposed targeting the connections between the CS and LA through surgery or ablation. In 1989, Guiraudon *et al.*^8^ developed a surgical procedure in which the atria are divided into three components: the LA, the RA, and a corridor from the sinus node to the atrioventricular node. This procedure was successful in 31 of 36 patients, with 69% free from arrhythmias at follow-up. However, pacemakers were needed in 16% of the patients, and it was difficult to isolate the LA from the corridor in the surgical procedure. These initial attempts to electrically isolate the atria were surgically challenging. Huo *et al.*^2^ recently presented the first reports of feasibility and electrophysiological outcome of a simplified LA isolation catheter ablation approach for patients with severe left atrial disease. At present, it is not clear which combination of electrical connections are important in atrial arrhythmogenesis, and which patients are likely to benefit from this procedure.

We tested the ability of IAC ablation to terminate sustained AF in a virtual patient cohort. IAC ablation was applied for each combination of the three connections, together with or without the application of PV isolation (PVI). We assessed the effects of patient-specific geometry, LA-to-RA frequency gradient, and fibrotic remodelling on the predicted efficacy. We hypothesized that IAC ablation is more likely to be successful and return the RA to SR in cases with LA drivers, but not for cases with RA drivers. To provide indicators of patients who may benefit from this procedure, we assessed arrhythmia cycle length (CL), arrhythmia driver location, atrial size, and fibrosis distribution for different IAC ablation outcomes.

## Methods

We describe first the model construction, including the anatomy, the interatrial structures, and the techniques used for incorporating atrial fibrosis in the model. We then outline the simulation details, including the arrhythmia pacing protocol and the types of ablation applied (PVI and IAC ablation). Finally, the analysis techniques used to classify the arrhythmia and its mechanisms are described, including phase singularity (PS) analysis.

### Atrial bilayer model

We constructed patient-specific atrial bilayer geometries from imaging data for patients with persistent AF. All patients gave written informed consent. This study is in accordance with the Declaration of Helsinki, and approved by the Institutional Ethics Committee at the University of Bordeaux. Late gadolinium enhancement magnetic resonance imaging (LGE-MRI) was performed using a 1.5T system (Avanto, Siemens Medical Solutions, Erlangen, Germany), with an average resolution of 0.625 mm × 0.625 mm × 2.5 mm. Imaging data were manually segmented using MUSIC software (Electrophysiology and Heart Modeling Institute, University of Bordeaux, and Inria, Sophia Antipolis, France, http://med.inria.fr). Atrial meshes were constructed from the segmented LA and RA endocardial surfaces. These surfaces were meshed using the Medical Imaging Registration Toolkit (mcubes algorithm), and cut using ParaView software (Kitware, Clifton Park, NY, USA) to generate openings at the mitral and tricuspid valves, the four PVs, the superior and inferior vena cava, and the CS. To produce meshes with resolutions suitable for simulation, remeshing was performed using mmgtools meshing software (http://www.mmgtools.org/), resulting in an average edge length of 0.34 mm to match the resolution of the original bilayer model.^9^

To assign atrial structures and fibre directions to the model, we used a technique to define the LA and RA of each patient in the same co-ordinate system, to transfer scalar and vector data from the previously published bilayer model. This technique was used to include endocardial RA structures in the model, including the pectinate muscles, the crista terminalis and the sinoatrial node (SAN). The SAN structure of the bilayer model was assigned to match recent experimental data, including an average size of 14.8 mm by 4.3 mm,^10^ with four connection points. The left atrium of the model was composed of linearly coupled endocardial and epicardial layers.

### Interatrial structures

We modelled the three major interatrial structures: BB, the CS, and the FO. These IACs were introduced through a combination of medical imaging data, augmented with anatomically informed automatically generated structures.

#### Bachmann’s bundle construction

Bachmann’s bundle is an interatrial structure that originates close to the junction of the superior vena cava with the RA body, connecting to the atrial fibres on the RA anterior wall, and then joining with the anterior wall of the LA, combining with the septopulmonary bundle that connects the FO to the left atrial appendage and PVs. It is approximated in the original bilayer model as a two-dimensional structure, generated from a three-dimensional manual segmentation of BB from computed tomography (CT) imaging.^9^

**Figure 1:**
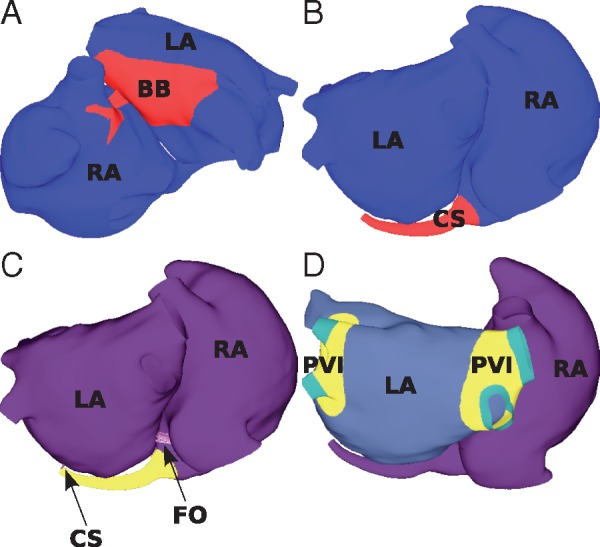
Interatrial connections. The LA and RA models are connected at (*A*) BB; (*B*) the CS; and (*C*) the FO. (*D*) To isolate the PVs, the tissue shown in yellow (labelled PVI) is set to non-conductive. The interatrial structures are connected to the LA and RA using discrete resistance line elements, shown as purple lines and indicated by the arrows in (*C*). BB, Bachmann’s bundle; CS, coronary sinus; FO, fossa ovalis; LA, left atrium; PVs, pulmonary veins; PVI, pulmonary vein isolation; RA, right atrium.

To incorporate the structure in patient-specific meshes from the MRI data used in this study, we used a combination of mapping from the original bilayer model, together with an automated mesh generation technique. Specifically, components of BB in close proximity to the LA and RA meshes were transferred from the atlas bilayer model^9^ using scalar mapping. Nodes in these components of BB were then electrically connected to the LA endocardial and epicardial surfaces, and RA epicardium using discrete resistance line connections to the closest nodes. Finally, the LA and RA components of BB were linked to each other to create a continuous structure (shown in *Figure 1A*) using a custom-written automatic mesh construction algorithm.

#### Coronary sinus construction

Part of the CS structure was available from the segmented LGE-MRI data, and this was then extended to join the RA mesh to the LA mesh, using a cylindrical structure, shown in *Figure 1B*. This was defined as following the morphology of the LA, with a degree of taper along the cylinder. The half of the distal CS rim closest to the LA body was joined to the closest LA nodes using discrete resistance line elements (shown in *Figure 1C*), in order to electrically connect the CS to the LA body.

#### Fossa ovalis construction

The left and right atrial meshes were joined at the interatrial septum, by electrically connecting the LA and RA FO rims (see *Figure 1C*).

### Tissue level and cellular level properties

Tissue conductivities in the model were tuned to agree with the human activation mapping data of Lemery *et al.*^5^ The Courtemanche–Ramirez–Nattel human atrial ionic model was used with modifications and regional heterogeneity in repolarization as in our previous studies.^11^^,^^12^ These modifications reproduce previously reported differences in repolarization properties across the atria, including a shorter action potential duration (APD) in the PVs,^13^ a longer APD in the CT,^14^ and a difference in APD between the LA and RA body.^15^^,^^16^ Specifically, different rapid delayed rectifier current (*I*_Kr_) conductances were included in the LA and RA body of the baseline model (LA: 0.047 pS/pF and RA: 0.0294 pS/pF) based on the experimental findings of Li *et al.*^17^ and following Aslanidi *et al.*,^18^ resulting in model SR APD values: LA 205 ± 11 ms and RA 236 ± 8 ms.

### Left atrial vs. right atrial frequency differences

Multiple clinical studies report higher dominant frequencies in the LA than the RA during AF.^19–21^ Similarly, arrhythmia simulations using the baseline model considered in this study have higher dominant frequencies (shorter AF CLs) in the LA than the RA. Lazar *et al.*^22^ reported the presence of higher AF frequency in the LA than the RA in paroxysmal AF patients, but equal AF frequencies in the LA and RA for persistent AF patients, suggesting that electrical remodelling may homogenize differences in repolarization between the LA and RA body.^16^ To test the effects of these changes in repolarization on IAC ablation outcome, the *I*_Kr_ conductance was set to the LA value for all regions of the model for a subset of the simulations.

### Modelling atrial fibrosis

Atrial fibrotic remodelling is multifactorial and the optimal way to incorporate remodelling in simulation studies is unclear. To account for this uncertainty, we considered two approaches for modelling atrial fibrosis: an interstitial type of fibrosis, and a combination type of remodelling that also incorporates cellular changes and modifications in the tissue level conductivity.

#### Interstitial fibrosis

In interstitial fibrosis simulations, fibrosis was modelled using a probabilistic approach, depending on both patient-specific LGE-MRI intensity value and the fibre direction. Edges of elements of the mesh were randomly selected as fibrotic with probability weighted by the LGE-MRI intensity and the angle of the edge compared with the element fibre direction, where edges in the longitudinal fibre direction were four times more likely to be selected than those in the transverse direction, following our previous methodology.^23^

#### Multifactorial combination fibrotic remodelling, incorporating conductivity, and ionic changes

In models where fibrosis was represented by a combination type fibrosis, we included conductivity changes and ionic level modifications associated with fibrosis, together with interstitial fibrosis. Conductivity changes followed Krueger *et al.*,^24^ with values representing mild, moderate, and severe fibrosis. For regions with 0–56% LGE intensity, conduction velocity (CV) was set to 100% of the baseline value. Mild, moderate, and severe fibrosis regions were then as follows: 56–60% LGE intensity, 80% CV; 60–64% LGE intensity, 60% CV; and >64% LGE intensity, 40% CV. Ionic changes to model the effects of TGF-β1 followed Zahid *et al.*^25^ with rescalings to modify maximal ionic conductances as follows: 50% gK1, 60% gNa, and 50% gCaL. These ionic changes were included in regions with LGE intensity >3 standard deviations (SD) above the blood pool mean, calculated as the average intensity of pixels in the range 0.02–40%, following Oakes *et al*.^26^

### Atrial fibrillation initiation protocol

Each model set-up was run for 1 s of no pacing, followed by 10 SR beats at a CL of 700 ms. Arrhythmia was then induced in the model using an extrastimulus pacing protocol from the right superior PV, using a clinically motivated protocol to simulate the occurrence of PV ectopics. The right superior PV was paced with five beats at a CL of 155–165 ms, with a coupling interval for the first PV beat following SR in the range 200–500 ms.^27^

### Simulation protocol

To test the effects of IAC ablation, together with and without the application of PVI, we used the following approach. For each set-up, AF was analysed (as detailed in Arrhythmia Analysis section) 3 s post-initiation, for 2 s. At 5 s post-initiation, ablation of IACs or PVI was applied. Arrhythmia dynamics were analysed for 2 s post-ablation. In the case of combined PVI and IAC ablation, IAC ablation was applied 2 s post-PVI, and again the arrhythmia dynamics were assessed for a further 2 s.

### Interatrial connection ablation and pulmonary vein isolation ablation

To simulate the ablation of each of the three interatrial structures, the conductivities of the discrete resistance line elements between the interatrial structures and the LA and RA were set to zero. These include the line elements between the rim of nodes at the left and right atrial FO; the line elements between BB and the LA endocardium, the LA epicardium and the RA epicardium; and the line elements between the CS and the LA body.

Pulmonary vein isolation ablation was automatically applied to each of the different atrial geometries (see *Figure 1D*).

Each of the three IACs can either be ablated or not, with or without the addition of PVI, to give 16 combinations, including the base case of no IAC ablation with no PVI.

### Simulations

Simulations were run for the 12 patient-specific geometries, with four set-ups: the baseline model to assess the effects of geometry alone, with the removal of the LA:RA frequency gradient, with interstitial fibrosis, and with combination remodelling. For each model, 16 ablation types were considered, resulting in a total of 768 simulations.

All simulations were performed using the Cardiac Arrhythmia Research Package simulator (https://carp.medunigraz.at/carputils/).

### Arrhythmia analysis

The outcome of each type of ablation on the simulated dynamics was classified as either fibrillation, macro-reentry, or termination for each of the LA and RA. These classifications were visual and as follows: *fibrillation* consists of multiple meandering complex wavefronts; *macro-reentry* consists of an organized re-entrant circuit; *termination* means the absence of arrhythmia. The outcome of total IAC ablation (ablation of BB, the CS, and the FO) is classified as a success in the case that RA arrhythmia terminates, and a failure if the RA arrhythmia sustains (either fibrillation or macro-reentry).

Arrhythmia dynamics were assessed using phase analysis to indicate regions of rotational activity and wavefront break-up. Phase was calculated for each computational node as the angle around the trajectory generated by plotting the Hilbert transform of the action potential signal against the original signal. Spatial maps of phase were then post-processed to identify PSs at which there is a 2π progression in phase in the surrounding points and the phase value is undefined, by calculating the topological charge.^28^^,^^29^ Phase singularities may indicate rotational activity, wavefront break-up, or conduction block. To assess the overall distribution of PSs, PS spatial density maps were calculated using previously published methods.^23^ These maps were then partitioned into LA and RA. The total RA PS count was divided by the total model PS count over both atria, to give the *RA PS density ratio*. Average arrhythmia CL was calculated as the average interval between timings of isophase values for each node of the mesh and then averaged for the LA and the RA. These measures were calculated from AF simulation data measured for 2 s immediately preceding ablation.

The total model tissue surface area was calculated for each of the LA and RA to test the effects of area on arrhythmia type. The fibrotic content of the tissue was computed as the proportion of tissue with LGE intensity >3 SDs above the blood pool mean for the LA and RA. Values are quoted as mean and standard deviation, and mean values are compared using unpaired *t*-tests.

## Results

We first investigated the effects of IAC ablation in 12 patient-specific atrial geometries, to assess the efficacy of the ablation strategy in the absence of structural remodelling (see Geometry Alone Affects Interatrial Connections Ablation Outcome section). We tested the effects of ablation of each of the IACs individually or in combination on arrhythmia outcome, together with and without PVI. We then evaluated the effects of features of the atrial substrate on these outcomes, by modifying the frequency gradient between the LA and RA (see Left Atrial -to-Right Atrial Frequency Gradient Affects Interatrial Connections Ablation Outcome section), and including fibrotic remodelling, modelled either as interstitial fibrosis (see Interstitial Fibrosis Decreases Interatrial Connections Ablation Success Rate section) or a combination type remodelling (see Combination Remodelling Decreases Interatrial Connections Ablation Success Rate section). We finally dissected the influence of each factor on outcome by comparing LA and RA arrhythmia CL, tissue area, tissue fibrotic content and arrhythmia rotational content for cases in which RA arrhythmia terminated (IAC ablation success) compared to sustained (IAC ablation failure) (see Right Atrium Phase Singularity Density Ratio Predicts Interatrial Connections Ablation Outcome section).

### Geometry alone affects interatrial connections ablation outcome

**Figure 2:**
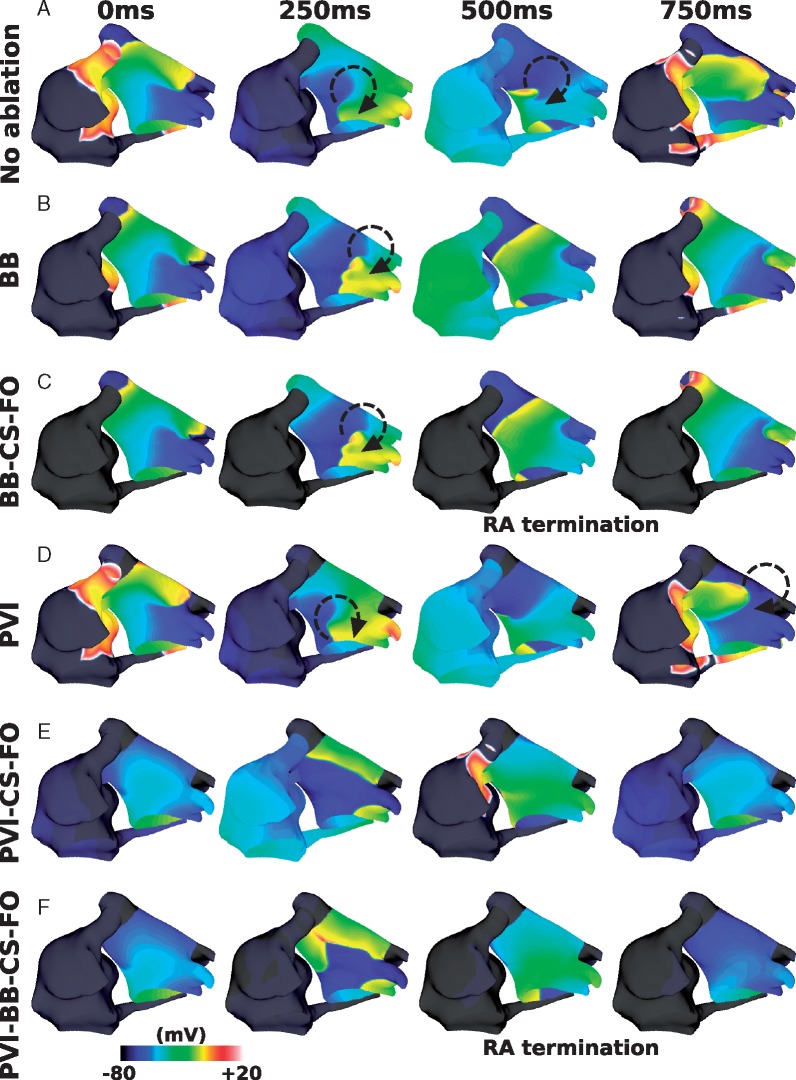
Response to different ablation therapies. Transmembrane potential plots for: (*A*) no ablation (LA AF and RA macro-reentry), (*B*) ablation of BB (LA AF and RA macro-reentry), (*C*) ablation of all three IACs (LA AF and RA termination), (*D*) PVI ablation (LA AF and RA macro-reentry), (*E*) PVI ablation together with CS and FO ablation (LA macro-reentry and RA macro-reentry), and (F) PVI ablation together with ablation of all three IACs (LA macro-reentry and RA termination). AF, atrial fibrillation; BB, Bachmann’s bundle; CS, coronary sinus; FO, fossa ovalis; IAC, interatrial connections; LA, left atrium; PVI, pulmonary vein isolation; RA, right atrium.

*Figure 2* shows an example of the response to different ablation approaches for one patient geometry. In this case, IAC ablation of all three connections (total IAC ablation) returns the RA to SR (*Figure 2C*), and this response is the same in the case where PVI is also applied (*Figure 2F*).

**Figure 3:**
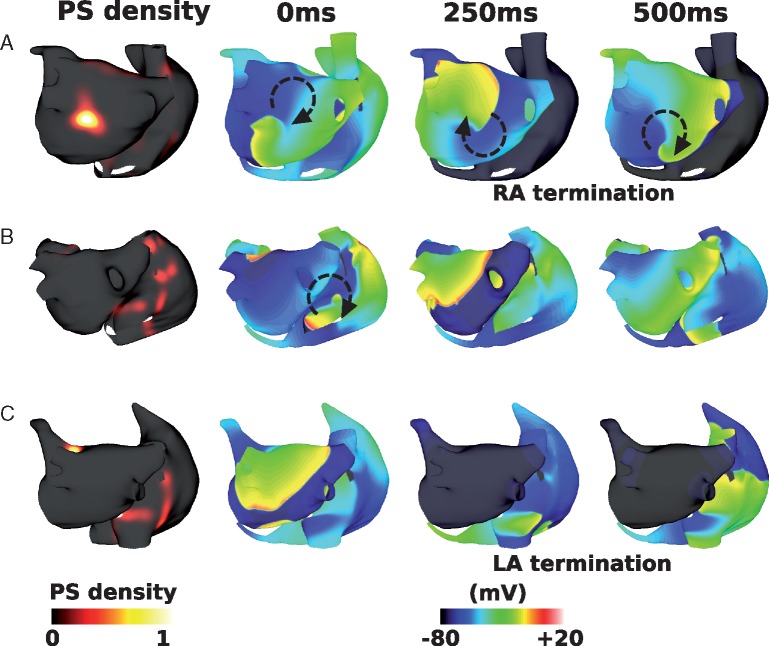
Geometry affects IAC ablation. (*A*) An example success (LA AF and RA termination); (*B*) an example failure (LA AF and RA AF); and (*C*) an example failure, with LA termination (LA termination and RA fibrillation). AF, atrial fibrillation; LA, left atrium; PS, phase singularity; RA, right atrium.

*Figure 3* shows that the response to IAC ablation varied across the different geometries. Total IAC ablation was successful in returning the RA to SR for 10 of the 12 geometries. An example of a successful IAC ablation is shown in *Figure 3A*. In the two geometries where IAC was unsuccessful, the arrhythmia was maintained in both the LA and RA in one case (*Figure 3B*) and only in the RA in the other (*Figure 3C*).

For nine of the 10 geometries for which total IAC ablation successfully terminated RA arrhythmia, ablation of all three connections was necessary for this outcome. For the remaining geometry, the model converted from fibrillation to a macro-reentry before ablation was applied, and IAC ablation of either CS or FO terminated the macro-reentry.

**Figure 4:**
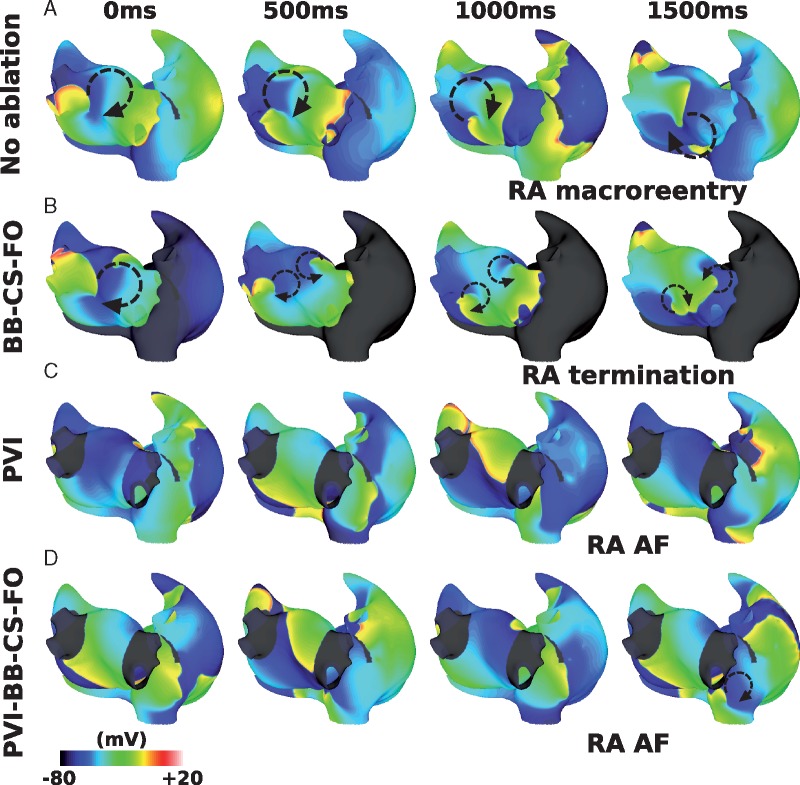
IAC outcome depends on whether or not PVI is applied. (*A*) Baseline model; (*B*) total IAC ablation; (*C*) PVI ablation; and (*D*) total IAC ablation after PVI ablation. Isopotential maps are at 500 ms intervals, with different start times to demonstrate the different responses. AF, atrial fibrillation; BB, Bachmann’s bundle; CS, coronary sinus; FO, fossa ovalis; IAC, interatrial connections; PVI, pulmonary vein isolation; RA, right atrium.

For one of the 10 geometries counted as IAC ablation success, the outcome changed if IAC ablation was instead applied post-PVI; whereas in all other cases, this did not affect the outcome. This case is shown in *Figure 4*, in which the RA activity for the baseline model exhibits passive activation, simply responding to the LA activity (*Figure 4A*). Right atrial arrhythmia termination occurs following total IAC ablation in *Figure 4B*. However, applying PVI in the model (*Figure 4C*), decreases the LA CL, and modifies the RA arrhythmia to a more complicated re-entry (classified as RA fibrillation), which is no longer simply passive to the LA activity. Applying IAC ablation following PVI, then results in arrhythmia in both the LA and RA (*Figure 4D*), with a macro-reentry around the junction between the PVs and the LA, and fibrillation in the RA.

The mean number of PSs across the baseline models was 2.4 ± 1.3 (LA: 1.8 ± 0.7 and RA: 0.6 ± 0.9). Mean LA CL was 182.9 ± 2.7 ms; this was similar for success and failure cases (success 183.7 ± 2.6 ms and fail 180.4 ± 1.5 ms). Mean RA CL was 226.2 ± 46.1 ms, and was longer in successful cases than failure cases but not significantly so (success 235.5 ± 50.2 ms and fail 198.2 ± 7.3 ms, unpaired *t*-test not significant). Mean LA surface area was 128.8 ± 23.0 cm^2^ (success 128.3 ± 23.3 cm^2^, fail 130.1 ± 16.8 cm^2^); mean RA surface area was 142.8 ± 29.2 cm^2^ and this was larger in IAC failure cases but not significantly so (success 134.5 ± 27.8 cm^2^, fail 167.7 ± 19.7 cm^2^, and not significant). Number of PSs in both the LA and RA was significantly larger for IAC failure cases than IAC success cases (LA success 1.5 ± 0.4 vs. fail 2.7 ± 0.8, *P* < 0.01 by unpaired *t*-test; RA success 0.3 ± 0.2 vs. fail 1.6 ± 1.3, *P* = 0.01). Mean RA PS density proportion was 0.18 ± 0.16, and this was larger for IAC failure cases (success 0.13 ± 0.12, fail 0.31 ± 0.23, and not significant).

### Left atrial-to-right atrial frequency gradient affects interatrial connections ablation outcome

Removing the LA-to-RA frequency gradient increased the likelihood that RA arrhythmia terminates after IAC ablation; RA arrhythmia terminated for all 12 geometries with the frequency gradient removed.

The mean number of PSs was similar to the baseline model (2.4 ± 0.6 compared with 2.4 ± 1.3). The change in *I*_Kr_ conductance decreased mean RA CL across the geometries from 226.2 ± 46.2 ms to 188.2 ± 8.6 ms, whereas mean LA CL was unaffected (182.9 ± 2.7 ms vs. 180.9 ± 1.4 ms). Despite this change in mean RA CL, the proportion of PS density in the RA of the model was similar to the baseline case: 0.18 ± 0.16 (baseline) vs. 0.20 ± 0.24 (no frequency gradient).

### Interstitial fibrosis decreases interatrial connections ablation success rate

**Figure 5:**
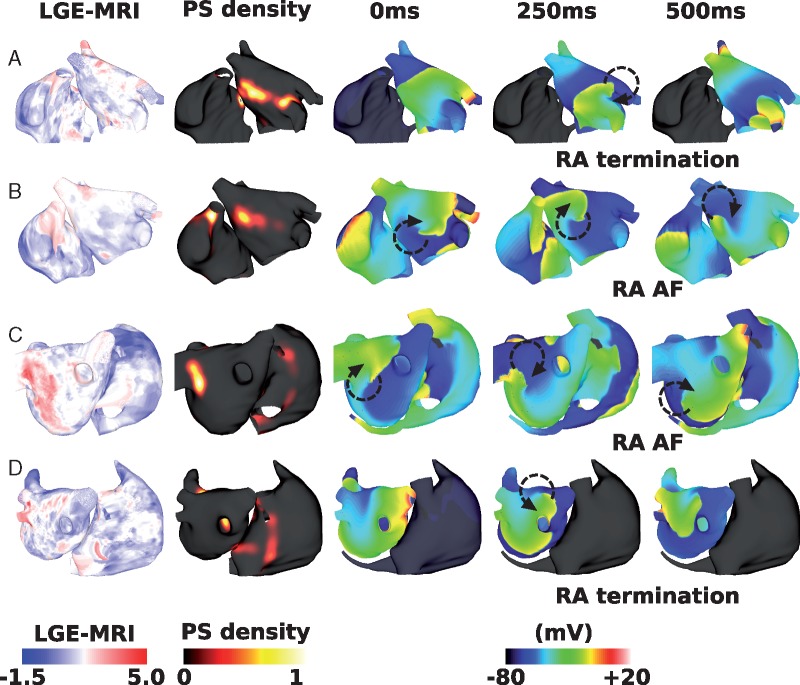
Effects of interstitial fibrosis on IAC ablation. Plots show LGE-MRI distributions, PS density distributions measured pre-IAC ablation, and transmembrane potential maps measured at three time points post-IAC ablation. (*A*) An example success, in which RA arrhythmia terminates. (*B*) An example failure, as RA arrhythmia sustains post-total IAC ablation. (*C*) An example failure. (*D*) An example success, as RA arrhythmia terminates. (*A*) and (B) are shown in anteroposterior view; (*C*) and (*D*) are shown in posteroanterior view. AF, atrial fibrillation; IAC, interatrial connections; LGE-MRI, late gadolinium enhancement magnetic resonance imaging; PS, phase singularity; RA, right atrium.

Including interstitial fibrosis in the model decreased the likelihood that IAC ablation terminated RA arrhythmia: RA arrhythmia terminated following total IAC ablation for 6 of the 12 geometries. *Figure 5* shows an example responses to total IAC ablation for four different geometries and LGE distributions, including cases where the RA arrhythmia terminated (*Figure 5A* and *D*), and where the RA arrhythmia sustained (*Figure 5B* and *C*).

The mean number of PSs was higher than the baseline model (3.7 ± 2.0 vs. 2.4 ± 1.3). Mean RA and LA CLs were the same with the addition of interstitial fibrosis as the baseline model (LA CL: 185.9 ± 5.5 ms; RA CL: 220.9 ± 49.7 ms). Mean LA CL was similar for success and failure cases (success 185.8 ± 5.6 ms and fail 186.0 ± 6.0 ms). Mean RA CL was longer in successful cases than failure cases but not significantly so (success 247.5 ± 75.6  ms, fail 203.2 ± 8.9 ms, and not significant). Mean LA surface area was larger for IAC failure cases (success 116.8 ± 25.7 cm^2^, fail 141.0 ± 18.4 cm^2^, and not significant); mean RA surface area was also larger in IAC failure cases (success 132.9 ± 23.9 cm^2^, fail 153.3 ± 32.8 cm^2^, and not significant).

The inclusion of interstitial fibrosis in the model increased the arrhythmia complexity, particularly in the RA. The number of PSs in the LA was similar between IAC success and failure cases (LA success 1.8 ± 0.2 vs. fail 2.2 ± 0.8); whereas for the RA the number of PSs was significantly larger for IAC failure cases than IAC success cases (RA success 0.7 ± 0.5 vs. fail 2.5 ± 1.7, *P* = 0.04 by one-sided unpaired *t*-test). Right atrial PS density ratio increased with the addition of interstitial fibrosis from 0.18 ± 0.16 to 0.46 ± 0.27, and this was larger for IAC failure cases (success 0.19 ± 0.20 and fail 0.64 ± 0.13, *P* < 0.001 by unpaired *t*-test). However, there was no correlation between LGE intensity and PS density for the interstitial fibrosis simulations (correlation 0.05 ± 0.03). Average fibrotic content was similar for successful and unsuccessful IAC ablation cases (LA fibrotic content: success 0.22 ± 0.07 and fail 0.24 ± 0.21; RA fibrotic content: success 0.12 ± 0.03 and fail 0.10 ± 0.10; total fibrotic content: success 0.18 ± 0.05 and fail 0.18 ± 0.16).

### Combination remodelling decreases interatrial connections ablation success rate

Including combination remodelling in the model set-up also decreased the likelihood that IAC ablation terminated RA arrhythmia, but to a lesser extent than for interstitial fibrosis; RA arrhythmia terminated following total IAC ablation for eight of the 12 geometries.

**Figure 6:**
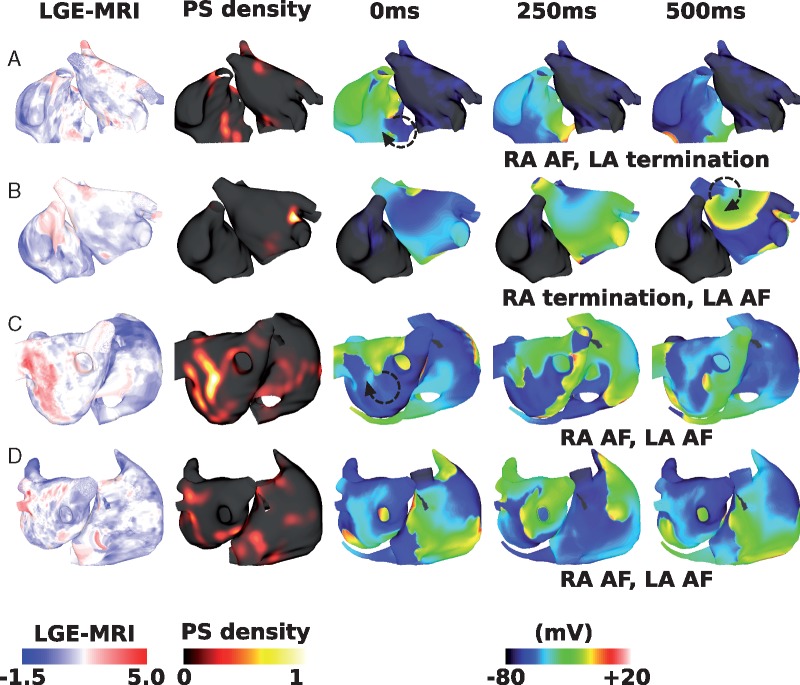
Effects of combination remodelling on IAC ablation. These maps correspond to the examples shown in *Figure 5*. (*A*) Example failure as RA AF sustains post-IAC ablation, and LA arrhythmia terminates. (*B*) An example success (compared with failure for interstitial case). (*C*) An example failure (same outcome as interstitial fibrosis). (*D*) An example failure (compared with success for interstitial case). AF, atrial fibrillation; IAC, interatrial connections; LA, left atrium; LGE-MRI, late gadolinium enhancement magnetic resonance imaging; PS, phase singularity; RA, right atrium.

*Figure 6* shows example responses for different geometries and LGE distributions, corresponding to the cases shown in *Figure 5*. The case shown in *Figure 5A* terminated following total IAC ablation for both fibrosis representations. The case shown in *Figures 5B* and *6B* exhibits RA AF following total IAC ablation for interstitial fibrosis, but not for combination remodelling. The case shown in *Figures 5C* and *6C* shows LA and RA AF following total IAC ablation for both representations of fibrosis. *Figures 5D* and *6D* are examples of RA termination for interstitial fibrosis, but RA arrhythmia for combination remodelling, following total IAC ablation.

Incorporating combination remodelling generates arrhythmias with a higher number of PSs than for the interstitial fibrosis models (combination remodelling mean: 7.7 ± 3.2; interstitial fibrosis mean: 3.7 ± 2.0). Adding combination remodelling prolongs both LA and RA mean CL (from 182.9 ± 2.7 ms to 230.4 ± 36.6 ms, and from 226.2 ± 46.1 ms to 236.9 ± 33.3 ms, respectively). Thus combination fibrotic remodelling acts to homogenize LA-to-RA gradients in CL. Mean LA CL was similar for success and failure cases (success 228.0 ± 49.8 ms and fail 233.4 ± 15.8 ms). Mean RA CL was significantly longer in successful cases than failure cases (success 256.2 ± 32.3 ms and fail 212.9 ± 13.5 ms, means significantly different by unpaired *t*-test *P* = 0.04). Mean LA surface area was similar for success and failure cases (success 126.6 ± 28.3 cm^2^ and fail 134.9 ± 23.5 cm^2^); similarly mean RA surface area was similar (success 144.3 ± 40.9 cm^2^ and fail 142.9 ± 20.6 cm^2^). The number of PSs in the LA was similar between IAC success and failure cases (LA success 4.3 ± 0.7 vs. fail 4.7 ± 1.3), whereas for the RA the number of PSs was significantly larger for IAC failure cases than IAC success cases (RA success 1.4 ± 1.4 vs. fail 5.5 ± 2.9, *P* = 0.03 by one-sided unpaired *t*-test). Mean RA PS density proportion was significantly larger than baseline at 0.35 ± 0.21, and this was larger for IAC failure cases (success 0.18 ± 0.10 and fail 0.54 ± 0.12, means significantly different by unpaired *t*-test *P* = 0.001).

In addition, PS locations show a much closer correspondence to the LGE intensity than for the interstitial fibrosis models (mean correlation 0.42 ± 0.06). In contrast to interstitial fibrosis in which average fibrotic content was similar for successful and unsuccessful IAC ablation cases, for combination fibrotic remodelling there is a difference (LA fibrotic content: success 0.23 ± 0.20 and fail 0.36 ± 0.14; RA fibrotic content: success 0.06 ± 0.04 and fail 0.12 ± 0.06; and total fibrotic content: success 0.15 ± 0.11 and fail 0.25 ± 0.09).

### Right atrium phase singularity density ratio predicts interatrial connections ablation outcome

**Figure 7:**
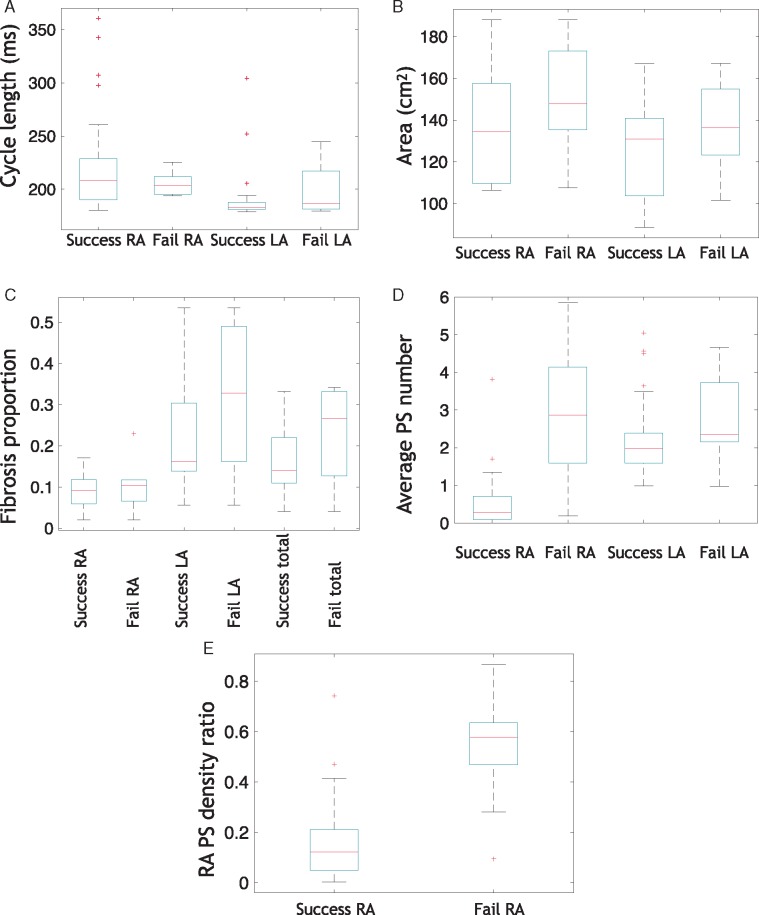
RA PS density ratio predicts IAC ablation outcome. Cases are classified as success or fail depending on whether RA arrhythmia terminates post-IAC ablation, and mean properties calculated for each of the LA and RA. (*A*) Average CL in the RA and LA, (*B*) average area, (*C*) proportion of the RA, LA, and total tissue assigned as fibrotic, (*D*) Average number of PSs in the RA and LA at a given time instance (means significantly different under unpaired *t*-test, with *P* < 0.0001 for the RA and *P* = 0.04 for the LA), (*E*) RA PS density ratio (means significantly different under unpaired *t*-test, with *P* < 0.0001). Boxes indicate 25th and 75th percentiles, the red lines indicate median locations, and crosses represent outliers. AF, atrial fibrillation; CL, cycle length; IAC, interatrial connections; LA, left atrium; PS, phase singularity; RA, right atrium.

To determine whether substrate properties, or arrhythmia properties pre-ablation correlate with IAC outcome, simulations were classified as successful or unsuccessful IAC ablation, depending on whether RA arrhythmia terminates or not. All four modelling set-ups were considered together for all 12 geometries, including simulations using the baseline model, without the LA: RA frequency gradient, with interstitial fibrosis and with combination remodelling. *Figure 7* shows the properties of successful and unsuccessful IAC ablations for the LA and RA, including CL, size, fibrotic content, PS number and PS density. Although the spread of RA CLs is smaller for failure cases (meaning that failure cases are more likely to be short RA CL), and LA CL spread is smaller for successful cases (meaning that successful cases are more likely to have a short LA CL), the differences in the means were not statistically significant (*Figure 7A*). Similarly, RA area is larger (*Figure 7B*) for failure cases, but this was not statistically significant. The proportion of the tissue classified as fibrotic is higher for failure cases, but not statistically significant (*Figure 7C*). The average number of PSs expected at a given time instance in the LA and RA (*Figure 7D*) is significantly higher for failure cases for both the LA and RA, and this difference is larger for the RA (LA success 2.2 ± 1.1 vs. fail 3.1 ± 1.5, means significantly different under unpaired *t*-test, with *P* = 0.04; RA success 0.6 ± 0.8 vs. RA fail 3.2 ± 2.5, *P* < 0.0001). Right atrial PS density ratio (*Figure 7E*) is significantly higher for failure cases than for successful cases (means significantly different under unpaired *t*-test, with *P* < 0.0001).

## Discussion

### Main findings

This *in silico* study demonstrates the use of a simulation platform for generating preliminary evaluation and guidance on patient selection for more speculative treatment approaches for persistent AF patients. Specifically, we tested ablation of IACs, to find that this is an effective treatment strategy in 75% of simulated cases (total IAC ablation applied across the 12 geometries for all four set-ups). Models without a LA-to-RA frequency gradient had an increased success rate, despite a shorter RA CL, suggesting this treatment will be more successful for persistent AF patients who have a smaller frequency gradient. Incorporating either interstitial fibrosis or combination fibrotic remodelling in the model increased arrhythmia complexity and reduced the success rate. For combination fibrotic remodelling, the success rate was lower for cases with high RA fibrotic content. Model PS density in the RA was higher pre-ablation in cases for which IAC ablation failed to terminate RA arrhythmia, suggesting this measure may inform whether IAC ablation is likely to be a suitable ablation strategy for a given persistent AF patient.

### Effects of atrial geometry on interatrial connections ablation

The response to IAC ablation depended on the atrial geometry (see *Figure 3*). The RA atrial size of the three cases in which RA arrhythmia sustained following PVI and total IAC ablation (147.7 cm^2^, 168.3 cm^2^, and 187.1 cm^2^) are larger than cases in which RA arrhythmia terminated (mean 134.4 cm^2^), while mean LA size is similar (130.1 cm^2^ vs. 128.3 cm^2^), meaning that larger RA size may indicate the likelihood of RA arrhythmia sustaining post-IAC ablation. This suggests that IAC ablation may not be a suitable treatment strategy for patients with large RA. However, RA atrial size was not significantly different between successful and unsuccessful IAC ablation simulations when all of the simulated cases were pooled, including those without a frequency gradient and with fibrosis (see *Figure 7B*). In particular, RA atrial size was similar between successful and unsuccessful IAC ablation simulations for cases with combination remodelling. For these models, RA fibrotic content is also important. This shows that this is not a simple relationship, and RA atrial size alone is not sufficient for predicting RA arrhythmia termination following total IAC ablation.

### Relationship between right atrial frequency and ablation outcomes

Under physiological conditions the RA and LA have different electrophysiological properties; during paroxysmal AF this results in a LA-to-RA frequency gradient (LA/PV junction: 6.2 ± 0.8 Hz, RA: 5.1 ± 0.6 Hz^22^); however, in persistent AF this frequency gradient disappears, potentially due to homogenization of electrophysiological properties.^22^ We investigated the effect of LA-to-RA frequency gradient on simulated IAC ablation outcome to find that removal of the gradient increased the likelihood of RA arrhythmia termination, with all models converting to SR following total IAC ablation, despite a decrease in RA CL (from 226.2 ms to 188.8 ms). This improved termination may be explained by the fact that the rate of activation of the RA from the LA is not fast enough to cause unidirectional block in the RA and develop into AF. Our results suggest that IAC ablation treatment may have increased efficacy in persistent patients in comparison to paroxysmal patients, in the case that the LA-to-RA frequency gradient disappears. This is important as IAC ablation therapy would likely only be used in more advanced persistent patients for whom other treatments have failed.

Hasebe *et al.*^30^ found a right-to-left dominant frequency (DF) gradient in a group of patients with RA-ectopy, and a left-to-right DF gradient in a group of patients with PV-ectopy. This suggests that this frequency gradient may indicate the location of atrial trigger sites. Hence, measurement of LA-to-RA frequency gradient may indicate atrial trigger sites and also whether IAC ablation is a suitable therapy.

### Inability of cycle length to stratify patients

Although a long RA CL indicates that total IAC ablation is likely to be a success, there are examples of both success and failure for short RA CLs, and there is no statistically significant difference in RA CL between success and failure cases (see *Figure 7A*). This implies that a low RA CL does not necessarily indicate that RA arrhythmia will sustain post-IAC ablation. For example, cases in which the frequency gradient was removed had a shorter RA CL, but IAC ablation was successful in all cases. Heterogeneity in repolarization is also likely to be important, and a ratio of CL to effective refractory period (ERP) may be an improved indicator.

### Effects of fibrotic remodelling on ablation outcome

Incorporating atrial fibrosis increased the arrhythmia complexity and decreased the likelihood that IAC ablation returned the RA to SR. Interstitial fibrosis alone caused more complex conduction (*Figure 5*), with more wave break, leading to a greater number of wavefronts in both the LA and RA. Right atrial arrhythmia increased due to both an increase in conduction heterogeneity and wavebreak in the RA, together with an increase in the variability of the interval between beats entering the RA from the IACs (due to an increase in LA conduction heterogeneity), which in turn increases the likelihood of a wavefront falling within the vulnerable window for unidirectional conduction block.

The combination fibrotic remodelling model in this study had the same distribution of interstitial fibrosis as the interstitial fibrosis model, with the addition of conductivity and ionic changes, following Krueger *et al.*^24^ and Zahid *et al.*,^25^ respectively. Interatrial connections ablation resulted in the RA returning to SR for more cases than for interstitial fibrosis alone (*Figure 6*). This may be because the conductivity changes and ionic changes slow conduction and increase ERP, leading to an increase in average wavelength, so that fewer arrhythmias are sustained. However, this is a complicated relationship, with cases with sustained RA AF following total IAC ablation for the combination fibrotic remodelling model, but not for the interstitial fibrosis model, and vice versa. This agrees with our previous study that showed that PS density depends on fibrosis methodology,^23^ but extends this to show that these differences also affect ablation outcome. For combination remodelling simulations, IAC ablation outcome corresponds with both RA and total fibrotic content. This suggests that IAC ablation is less likely to be successful for patients with advanced RA fibrosis. Late gadolinium enhancement magnetic resonance imaging or voltage mapping could be used to assess atrial fibrosis prior to attempting IAC.

### The effects of pulmonary vein isolation ablation

For one case, shown in *Figure 4*, applying PVI ablation before IAC ablation changed the outcome of total IAC ablation. This is important to consider for treatment planning, as procedures typically start with PVI. The effects of PVI on RA activity and arrhythmia are not typically considered, but this example shows that PVI may convert passive RA activity to fibrillatory activity in the RA. This happens because pre-PVI, many of the CS activation wavefronts are blocked in the refractory RA. Applying PVI increases the LA CL, such that activation from the CS no longer blocks in the RA, which in turn decreases the RA CL, leading to unidirectional block and re-entry. Pulmonary vein isolation ablation to eliminate PV triggers has the potential to terminate LA fibrillation, and so should be included in any IAC ablation therapy, but it is important to acknowledge that PVI may affect RA arrhythmia and IAC success rate.

### Predicting arrhythmia type and interatrial connections outcome

Both the mean number of PSs in the RA and the RA PS density to total PS density ratio are significantly higher for cases in which total IAC ablation fails to return the RA to SR (see *Figure 7D* and *E*). This suggests that IAC ablation is a less suitable strategy for patients in whom drivers are located in the RA. These could be cases in which the RA arrhythmia drives the LA, or cases with both LA and RA drivers. The mean number of PSs in the LA was also significantly higher for IAC failure cases, although the magnitude of this difference is much smaller; this difference likely reflects the increased likelihood of failure for more complicated arrhythmia substrates. Both mean number of PSs and the ratio of RA PS density to total PS density could be measured using a basket catheter in each atria, using the ECGi mapping technology, or using sequential multipolar catheter mapping—provided there is a degree of temporal stability in PS number and location—or alternatively predicted using patient-specific simulations. Using mean number of PSs in the RA alone requires measurements from the RA only, and so may be a more practical parameter to measure clinically.

For cases in which the CS is the primary IAC pathway, CS catheter electrogram recordings could indicate the direction of activation in the CS, and whether activation is in the LA to RA direction, or vice versa. In cases where AF activation is RA to LA, IAC ablation is less likely to return the RA to SR, as drivers for the fibrillation are likely to be in the RA.

Although RA CL is not significantly longer when all of the simulation results are pooled (*Figure 7A*), it is longer for the models with geometry alone, for interstitial fibrosis and for combination remodelling. Right atrial CL is much easier to measure than RA PS number because it can be recorded using any type of catheter and a small number of sites should suffice. Estimates of RA CL can be taken as the average interval between activation times on an electrogram, or using dominant frequency analysis to assess the frequency content of the signal. Hence, we recommend using this parameter, for which a longer RA CL indicates IAC ablation is more likely to be successful, while a short RA CL should be interpreted together with other information—for example, the RA PS density ratio. Right atrial area should also be considered in cases with less advanced fibrotic remodelling.

Patient-specific predictions could aid in cases where basket catheters or the ECGi technology are not available for phase mapping, as well as for pre-procedural treatment planning. Computational modelling incorporates information on atrial size, fibrosis distribution and ERP to predict arrhythmia dynamics and ablation outcome, which is important as the relationship between these factors and predicted IAC outcome is not simple. Biatrial bilayer models with patient-specific geometry and fibrosis distribution may be constructed from LGE-MRI imaging performed several weeks before the ablation procedure, and simulations of AF and IAC ablation run for set-ups with a range of different repolarization properties, to generate a family of outcomes. During the ablation procedure, LA and RA CLs (or fibrillation frequencies) could then be measured at multiple locations using standard catheters for electrogram recordings, and the simulation results for the experiment with the closest distribution of CLs used as a patient-specific prediction. This is one approach to circumvent the challenge of calibrating and running simulations during a single ablation procedure.

### Model construction: uncertainties and unknowns

#### Interatrial connections construction

Our computational model incorporates BB and the CS as separate atrial structures, and the septum of the left and right atria are modelled separately, with connection at the FO. As such, conduction between the atria of the model must pass through these structures, which are too narrow to support the transition of rotors between the LA and RA. Models with a combined LA and RA septal region might allow rotor drift between the atria. We do not know whether rotors drift between the LA and RA in human AF. Appropriate modelling of IACs is important to predict arrhythmia dynamics and response to ablation therapy. Patient-specific measurements of biatrial SR activation patterns, including characterization of IAC conduction, will improve understanding of atrial electrophysiology and function.

#### Atrial fibrosis

The AF substrate typically consists of both electrical remodelling and structural remodelling, including changes associated with fibrosis. The relationship between LGE intensity and cellular and tissue level fibrotic remodelling is unknown. As such, we considered two types of fibrosis representation, to test the effects of fibrotic remodelling on IAC ablation outcome, and to evaluate whether the type of fibrotic remodelling affects this outcome.

PS distributions from the interstitial model of fibrosis show a much lower correspondence with LGE intensity than the combination representation of fibrotic remodelling (compare *Figures 5* and 6). There is varied clinical evidence on the relationship between LGE intensity and rotational activity,^31^^,^^32^ and clinically observable differences could also reflect differences in the type of atrial remodelling. Further developments in the field on how to model atrial fibrosis are vital for ablation outcome prediction.

### Clinical challenges associated with total interatrial connections ablation

Although this simulation study suggests that catheter ablation of IACs may be a promising clinical approach for returning the RA to SR, this technique may be clinically difficult to achieve, particularly because of differences in IAC morphology across patients. Variability in the locations and numbers of connection points between the CS and LA mean that ablation or surgical approaches to remove CS-LA conduction is challenging. This may explain some of the problems associated with LA isolation by Guiraudon’s corridor^8^ or the Cox Maze procedure, and is likely to also be a problem associated with IAC ablation. The very recent clinical studies of Huo *et al.*^2^ apply a simplified catheter ablation approach for LA isolation, by including an anterior line, a paraseptal line, and circular right PVI to separate LA from RA. They demonstrate safety and feasibility for patients with advanced LA substrates.^4^ Their ablation approach may be clinically simpler than individually targeting the IACs.

### Pulmonary vein isolation ablation therapy and the need for new treatment approaches

While PVI is often an effective therapy for paroxysmal AF, it is less likely to work in patients with advanced AF; for example, the recent STAR AF II trial found that 41% of patients treated for persistent AF with PVI had recurrent AF 18 months after ablation therapy.^1^ Patients with advanced AF are the patient group most likely to benefit from IAC ablation, as they have few alternatives.

### Limitations

Our computational model has inherent assumptions. Left atrial and RA conductivities were tuned to the average data of Lemery *et al.*^5^ and were the same for all model geometries. Adopting recently proposed techniques for characterizing electrophysiological heterogeneity^33^ or personalizing models to ECGi data may address this limitation. Different RA-LA conduction patterns will affect AF dynamics and maintenance. In particular, improved electrical characterization of interatrial conduction patterns will improve patient-specific arrhythmia modelling. The CS and BB were incorporated in the model using a sequence of rules, rather than directly from imaging data. We tested the effects of ablation on pre-existing arrhythmia, but did not evaluate inducibility post-ablation. Further inducibility testing is required to assess the likelihood of AF re-initiation in the RA after IAC ablation causing AF recurrence.

## Conclusion

This simulation study predicts that IAC ablation is effective for many cases in returning the RA to SR. Patient-specific modelling approaches have the potential to stratify patients prior to ablation by predicting if AF drivers are likely to be located in the LA or RA.
